# *In Vitro* Wound Healing Potential and Identification of Bioactive Compounds from *Moringa oleifera* Lam

**DOI:** 10.1155/2013/974580

**Published:** 2013-12-31

**Authors:** Abubakar Amali Muhammad, Nur Aimi Syarina Pauzi, Palanisamy Arulselvan, Faridah Abas, Sharida Fakurazi

**Affiliations:** ^1^Laboratory of Vaccines and Immunotherapeutics, Institute of Bioscience, Universiti Putra Malaysia, 43400, Serdang, Selangor, Malaysia; ^2^Department of Food Science, Faculty of Food Science and Technology, Universiti Putra Malaysia, 43400, Serdang, Selangor, Malaysia; ^3^Laboratory of Natural Products, Institute of Bioscience, Universiti Putra Malaysia, 43400, Serdang, Selangor, Malaysia; ^4^Department of Human Anatomy, Faculty of Medicine and Health Sciences, and Laboratory of Vaccines and Immunotherapeutics, Institute of Bioscience, Universiti Putra Malaysia, 43400 Serdang, Selangor, Malaysia

## Abstract

*Moringa oleifera* Lam. (*M. oleifera*) from the monogeneric family *Moringaceae* is found in tropical and subtropical countries. The present study was aimed at exploring the *in vitro* wound healing potential of *M. oleifera *and identification of active compounds that may be responsible for its wound healing action. The study included cell viability, proliferation, and wound scratch test assays. Different solvent crude extracts were screened, and the most active crude extract was further subjected to differential bioguided fractionation. Fractions were also screened and most active aqueous fraction was finally obtained for further investigation. HPLC and LC-MS/MS analysis were used for identification and confirmation of bioactive compounds. The results of our study demonstrated that aqueous fraction of *M. oleifera* significantly enhanced proliferation and viability as well as migration of human dermal fibroblast (HDF) cells compared to the untreated control and other fractions. The HPLC and LC-MS/MS studies revealed kaempferol and quercetin compounds in the crude methanolic extract and a major bioactive compound Vicenin-2 was identified in the bioactive aqueous fraction which was confirmed with standard Vicenin-2 using HPLC and UV spectroscopic methods. These findings suggest that bioactive fraction of *M. oleifera* containing Vicenin-2 compound may enhance faster wound healing * in vitro. *

## 1. Introduction

Skin is one of the largest organs in human body and serves as a protective barrier to external noxious agents including microorganisms. This protective barrier could be disrupted due to injuries or wounds, hence, the need to maintain its structural integrity. Wound results in the loss of continuity of epithelium in the skin with or without the loss of underlying connective tissue [1]. Wound healing is a complex process of restoring impaired cells and tissues back to their normal state and occurs as a cellular response to injury and involves activation of fibroblast, endothelial cells, and macrophages [2]. Wound healing also involves a well-orchestrated integration of biological and molecular events of cell migration, cell proliferation, and extracellular matrix (ECM) deposition [3]. During wound healing process, growth factors released from fibroblasts, macrophages, neutrophils, keratinocytes, and endothelial cells influence all phases of wound healing and occur by providing signals for various cellular activities [4]. Management of chronic wound involves the use of antibiotics, anti-inflammatory agents, or combination of both, but some of these drugs are associated with unwanted side effects [5] hence the need for other alternatives without producing toxicity.

*Moringa oleifera* Lam is the most widely cultivated species of a monogeneric family *Moringaceae. *It is native to India, Pakistan, Bangladesh, and Afghanistan, utilized by ancient Romans, Greeks, and Egyptians; now distributed in many countries of the tropics and subtropics [6]. The edible parts of plant have been employed traditionally for skin diseases, rheumatism, anemia, cholera, and other ailment. The plant has an impressive range of medicinal uses with high nutritional value and serves as a good source of proteins, vitamins, beta-carotene, amino acid, and various phenolics [7]. It was reported to possess antimicrobial action [8], anti-inflammatory properties [9], antidiabetic [10, 11], antioxidant [12], and anticancer properties [13, 14]. It has also been reported to possess hepatoprotective activity through antioxidant nature [15–17]. Evaluation of various plant products according to their traditional uses and medicinal value based on their therapeutic efficacy leads to the discovery of newer and cost-effective drugs for treating various ailments. This forms the basis for selection of *M. oleifera* as a potential wound healing agent. To the best of our knowledge, the *in vitro* wound healing potential of this plants extracts or bioactive compounds has not been reported previously. Therefore, this study was aimed at exploring the potential of *M. oleifera *in enhancing wound healing and identification of putative bioactive compounds responsible for the wound healing action through *in vitro* process.

## 2. Materials and Methods

### 2.1. Chemicals and Reagents

All chemicals used were of analytical and HPLC grade (Merck, Germany). Methanol, ethanol, n-hexane, dichloromethane, ethyl acetate, and n-butanol were used for extraction and separation of compounds. Formic acid (Fisher, Loughborough, UK) was used as buffer. Cell culture fibroblast medium, fetal bovine serum, antibiotics, trypsin, trypsin neutralizing solution, MTT powder, and PBS, were all from Science Cell Research Laboratories (Carlsbad, USA). Commercial standard Vicenin-2 and bioactive compound were purchased from Haihang industrial company, Beijing, China. C_18_ column (3 *μ*m, 150 mm × 2.1 mm) was used for the separation and served as the stationary phase, while methanol and water were used as mobile phase.

### 2.2. Preparation of Plant Material and Extraction

The leaves of *M. oleifera *Lam (MO) were collected from Serdang City, Selangor, Malaysia, and were authenticated by Mr. Abdul-Ghaffar Othman from the Farm Unit of Faculty of Agriculture, Universiti Putra Malaysia (UPM), in October 2010. The air-dried powdered leaves of *M. oleifera* (500 g) were extracted successively with different solvents consisting of methanol, ethanol, and water to determine the most active solvent crude extract *in vitro* (at least three times for each solvent until the extract was exhausted) with the help of a shaker (Lab Tech shaker, model; LSI-3016R, Korea) set at 25°C. The extracts were then filtered and evaporated through rotary evaporator at 25°C (Buchi, Switzerland) to dryness under reduced pressure and further freeze dried with freeze drier (Virtis Bench Top K, United States) and the yield of each dried extracts was calculated and stored at −20°C until required for further use.

### 2.3. Differential Fractionation of the Crude Extract of Methanol

The active methanolic extract was chosen based on the *in vitro* screening results which consisted of *in vitro* wound scratch test assay and fibroblast proliferation assays in which all the three solvent crude extracts were screened to get the most active solvent crude extract, the bioactive methanolic crude extract, then was further subjected to differential bioguided fractionation using the *in vitro *screening procedures, and based on the results, aqueous fraction from the crude methanolic extract was the most active fraction. The most active methanolic crude extract was subjected further to differential fractionation using n-hexane, dichloromethane, ethyl acetate, n-butanol, and water. The methanoliccrude extract was redissolved in methanol : water (25 : 75) and partitioned against equivalent volume each of n-hexane, dichloromethane, ethyl acetate, and n-butanol and five fractions were obtained with respective solvents. The fractions were freeze dried and stored appropriately in −20°C until required for further study [18, 19].

### 2.4. HPLC Analysis

HPLC-DAD qualitative analyses were performed to determine and compare the general profiling of the compounds present in the most active crude extract of methanol and its aqueous fraction using Agilent Technologies (Santa Clara, USA) attached to a computer system. Gradient elution was carried out with methanol (solvent A) and water (solvent B) with 0.1% formic acid. C_18_ column was used for separation of active compounds at a flow rate of 0.8 mL/min. The extracts and fractions were prepared at concentration of 10,000 **μ**g/g of the sample injection volume being 10 **μ**L. The gradient program commenced from 5 : 95 (v/v) until 100 : 0 (v/v) of A : B over 65 min. UV detector was set 254 nm and the peak areas were generated automatically by computer using Agilent software.

### 2.5. LC-MS/MS Analysis

Mass spectra were acquired using Thermo Finnegan model LCQ (San Jose, CA) ion trap mass spectrometer equipped with an ESI source. The instrument was coupled to a surveyor diode array detector (DAD) (200–600 nm; 5 mm band width) and surveyor autosampler. The hyphenated system was supported with an X caliber 1.2 and mass frontier 5.0 software. Analytes separation was carried out on a Hipersil Gold C_18_ column (3 *μ*m, 150 mm × 2.1 mm) with a gradient mobile phase containing methanol (solvent A) and water (solvent B), each containing 0.1% formic acid. The gradient program commenced from 10 : 90(v/v) to 100 : 0 (v/v) of A : B over 65 mins with a flow rate of 250 *μ*L/min. The –ve ion mass spectra were obtained from LCQ DECA ESI/MS detector on full scan mode (50–1000 amu) at a scan rate of 0.5 Hz and the capillary temperature was set to 275°C. A data dependent program was used in the liquid chromatography-tandem mass spectrometry analysis, so that the most abundant ions in each scan were selected and subjected to MS/MS analysis. The collision induced dissociation (CID) energy was adjusted to 35% [19].

### 2.6. Quantification and Confirmation of Bioactive Compound Vicenin-2

#### 2.6.1. HPLC Method

HPLC was performed on a Jasco HPLC (Tokyo, Japan) consisting of a Jasco pump (PU-2086 Plus) and a Jasco UV detector model UV-2077 Plus linked by Jasco LC-Net II/ADC interface box and a software attached to a computer system. Analytical system was carried out on a C_18_ column. One mg of aqueous fraction of *M. oleifera* was weighed and dissolved in 1 mL 50 : 50 methanol : water and sonicated to ensure complete dissolution, this was then filtered through 0.45 *μ*m membrane filter paper (Whatman, GE Healthcare). The reference compound Vicenin-2 was also equally weighed and dissolved similar to aqueous fraction. Samples were stored at 4°C prior to analysis.

HPLC separation/confirmation of standard Vicenin-2 and aqueous fraction was performed at 254 nm. The flow rate and injection volume of the samples were 0.8 mL/min and 20 *μ*L. The chromatographic peaks of the analytes were confirmed by comparing their retention time and UV spectra with those of the reference standards Vicenin-2. The analysis was carried out at temperature of 25°C.

#### 2.6.2. UV/Vis Spectroscopic Analysis

The absorption spectrum of a sample can be measured as a plot of the absorbance of a sample as a function of wavelength. This plot can be used to identify an unknown sample since some compounds have characteristic absorption spectra. The UV-Vis spectroscopic measurements were made with a double-beam spectrophotometer (Lambda 35; Perkin Elmer) to obtain maximum absorption spectrum. The spectra were recorded between 220 and 600 nm. For the sample preparation, approximately 3 mL of the standard Vicenin-2 contains 0.003 M in distilled water and sample containing 0.003 M of aqueous fraction in distilled water placed in a 10 mm cuvette. In this experiment, we used distilled water as a reference.

### 2.7. *In Vitro* Biological Experiment

#### 2.7.1. *In Vitro* Screening of Different Solvent Crude Extracts and Fractions

*Cell Culture*. Human dermal fibroblast (HDF) cells were obtained from American Type Culture Collection (ATCC, Manassas, VA, USA, CRL-2301) and thawed as well as maintained according to the ATCC protocol [20]. Cells were cultured in fibroblast media premixed with 2% fetal bovine serum (FBS), growth supplements, and antibiotics consisting of L-glutamine 15 mmol/L, streptomycin 100 *μ*g/mL, and penicillin 100 U/Ml and incubated in 5% CO_2_ and 37°C. Cells at 80%–90% confluence were used for seeding and treatment throughout the experiment.

#### 2.7.2. (3-(4,5-Dimethylthiazol-2-yl)-2,5-diphenyl Tetrazolium Bromide (MTT)) Colorimetric Assays [21]

HDF cells were seeded into 96-well plate in triplicate at a density of 5 × 10^5^ (in 100 *μ*L medium) per well and grown for 24 hrs. The medium was replaced with serial dilutions of aqueous fraction of the crude methanolic extract at 12.5, 25, 50, 100, 200, 400, and 800 *μ*g/mL and the plates were incubated for 72 hrs. 10 *μ*L of MTT reagent was then added to each of the wells and incubated for another 3 hrs. The purple formazan formed was solubilzed by adding 100 *μ*L dimethyl sulfoxide (DMSO) to all the wells including control (without any treatment), then swirled gently to mix well and this was then kept in the dark place at room temperature for about 30 mins. ELISA microplate reader was used to read absorbance at 570 nm with reference of 630 nm. Graph of absorbance against number of cells was plotted to determine the HDF cells proliferation as per the standard methods.

#### 2.7.3. Fibroblast Proliferation and Viability Assay

Briefly, HDF cells were seeded on 24-well plates in triplicates and incubated at 37°C until confluent, and serial dilutions of each crude extracts (methanol, ethanol, and water) were added to cells and without any treatment served as control incubated for 24 hrs. The confluent of HDF cells were then released from the wells with trypsin and trypsin neutralizing solution (TNS) to count the cells with a hemocytometer. Typan blue dye (0.4%) was used for staining to differentiate between dead and viable cells because is a convenient, reliable, and inexpensive assay that makes faster distinction between dead and live cells [22]. The procedure was also repeated at 48 and 72 hrs. The number of cells obtained from the count corresponds to *n* × 10^4^ cells per milliliter of suspension. Experiments were performed in triplicate, and each sample was counted twice and the average reading was taken. Data were recorded and analyzed statistically using SPSS version 17.0 from IBM.

#### 2.7.4. Wound Scratch Assay Test

This experiment was performed according to the previously reported and standardized protocol [23]. HDF cells were seeded in a 24-well plates at a concentration of 3 × 10^5^ cells/mL cultured in a fibroblast media containing 5% FBS and grown to confluent cell monolayer. The media were pipetted out and discarded, a small area was then scratched using 200 *μ*L pipette tip, and the cells were then rinsed with PBS to remove the loosen debris of the cells. Fibroblast media with serial dilution of different concentration of methanolic extract at 3.125, 6.25, 12.5, 25, 50, and 100 *μ*g/mL were replaced and the plates were incubated at 37°C and 5% CO_2_. The distance between two layers of cells which was scratched by pipette tip was then inspected microscopically at 0, 24, 48, and 72 hrs, respectively. As the HDF cells migrate to fill the scratched area, and images were captured digital camera attached to microscope and computer system. The experiments were performed in triplicate and data were analyzed using Corel draw graphics suite x6 software.

### 2.8. Screening of Different Fractions from Crude Extract of Methanol

A bioguided assay fractionation was performed in order to obtain the most active fraction that could be further analyzed using HPLC and LC-MS methods to identify the bioactive compounds present in the fraction. A total of five fractions which consisted of n-hexane, dichloromethane, ethyl acetate, n-butanol, and aqueous were obtained from the differential fractionation of the most active methanolic crude extract through *in vitro* screening. Further characterization of five fractions was conducted through *in vitro *screening method as mentioned earlier, and the most bioactive fraction was identified.

### 2.9. Statistical Analysis

The data were analyzed by one way analysis of variance (ANOVA) using SPSS version 17.0 from IBM. Differences in mean between observations were considered significant at *P* < 0.05.

## 3. Results

### 3.1. Percentage Yields of Extracts and Fractions

**Figure 1:**
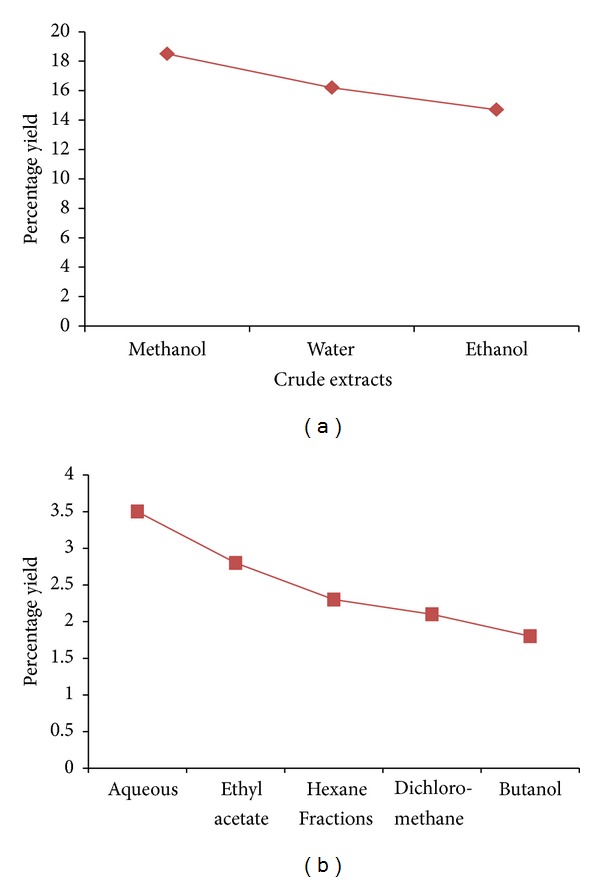
Percentage of yield of crude extracts and fractions of *M. oleifera* obtained following complete extraction of *M. oleifera *leaves. DCM: dichloromethane; EA: ethyl acetate. Values were obtained per 100 g of powdered leaves sample for the crude extracts and 10 g of dried methanolic extract for the aqueous fraction.

Quantitative estimation of the percentage crude extracts and fractions of *M. oleifera* showed that the yield of methanolic crude extract was highest (18.6%) and ethanolic crude extract was lowest (14.5%) per 100 g of powdered leaves of *M oleifera* (Figures 1(a) and 1(b)). Following bioguided fractionation of crude methanolic extract, aqueous fraction had a higher percentage yield of 2.5% compared to hexane with 1.8%, dichloromethane 1.6%, ethyl acetate 2.0%, and butanol 1.5%.

### 3.2. Effect of Different Crude Extracts on HDF Proliferation and Viability

**Figure 2:**
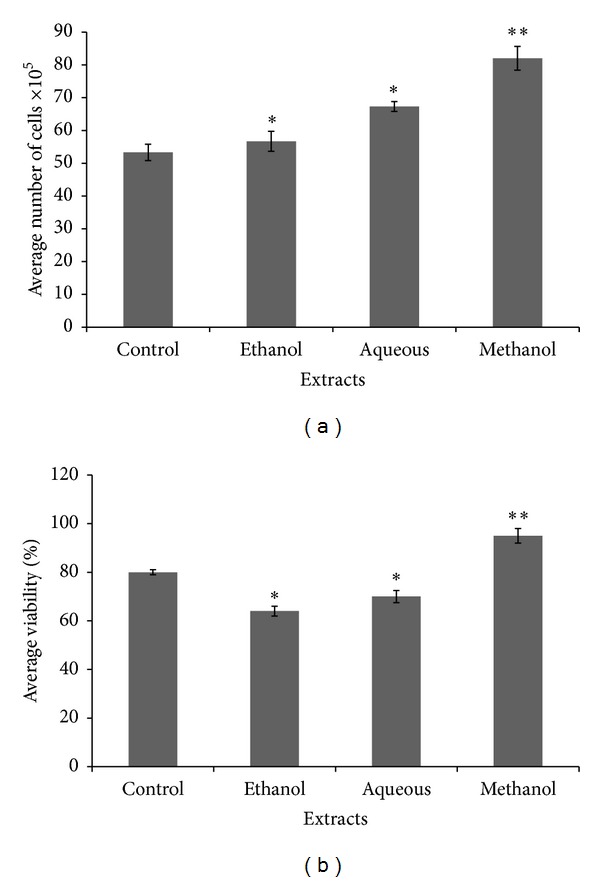
Effect of different crude extracts of *M. oleifera* on cell count and viability of human dermal fibroblast (HDF) administered at 12.5 *μ*g/mL. The data were expressed as mean ± SD of triplicate values. ANOVA was used for statistical analysis. **P < 0.05 (methanolic extract treated versus other solvent extracts and control).

**Figure 3:**
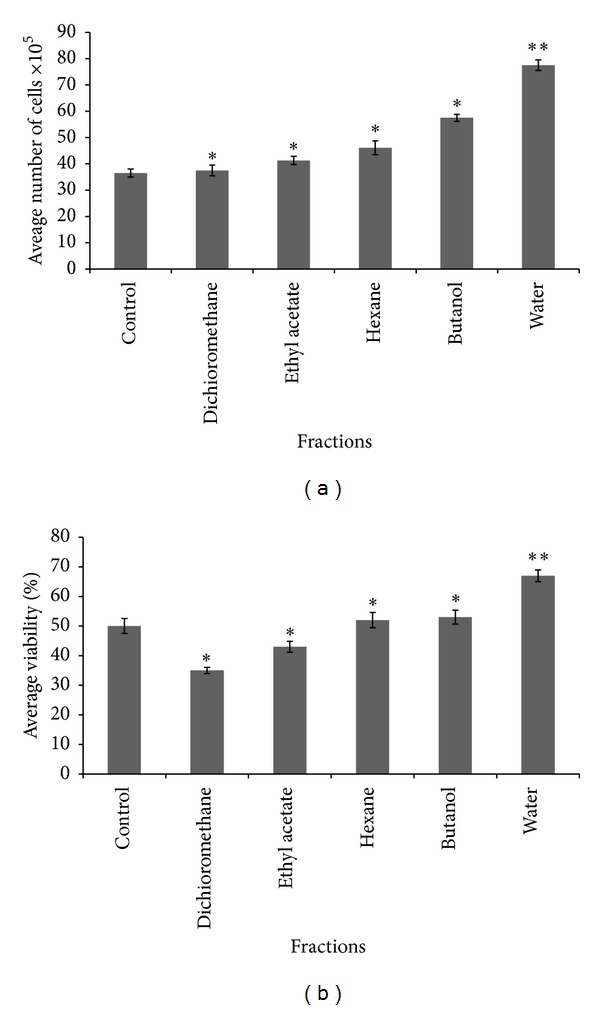
Effects of different fractions of *M. oleifera* on cell count and percentage viability of HDF administered at 25 *μ*g/mL of each fraction and readings were taken after 72 hrs. Percentage viability was estimated using standard formula as % cell viability = total viable cells/total cells (dead + viable) × 100. Data were expressed as mean ± SD of triplicate values. **P < 0.05 (methanolic extract versus other solvent extracts and control).

Cell proliferation as represented in Figures 2 and 3 shows significant difference in the rate of proliferation and viability (cell numbers) on HDF as expressed by the number of cell counts and cell morphology. In each set of experiments, methanolic crude extract and its corresponding aqueous fraction treated cells showed predominant number of cells and more stimulation of HDF cells that was significant from other extracts and control (*P* < 0.05).

### 3.3. Wound Scratch Test Assay

**Figure 4:**
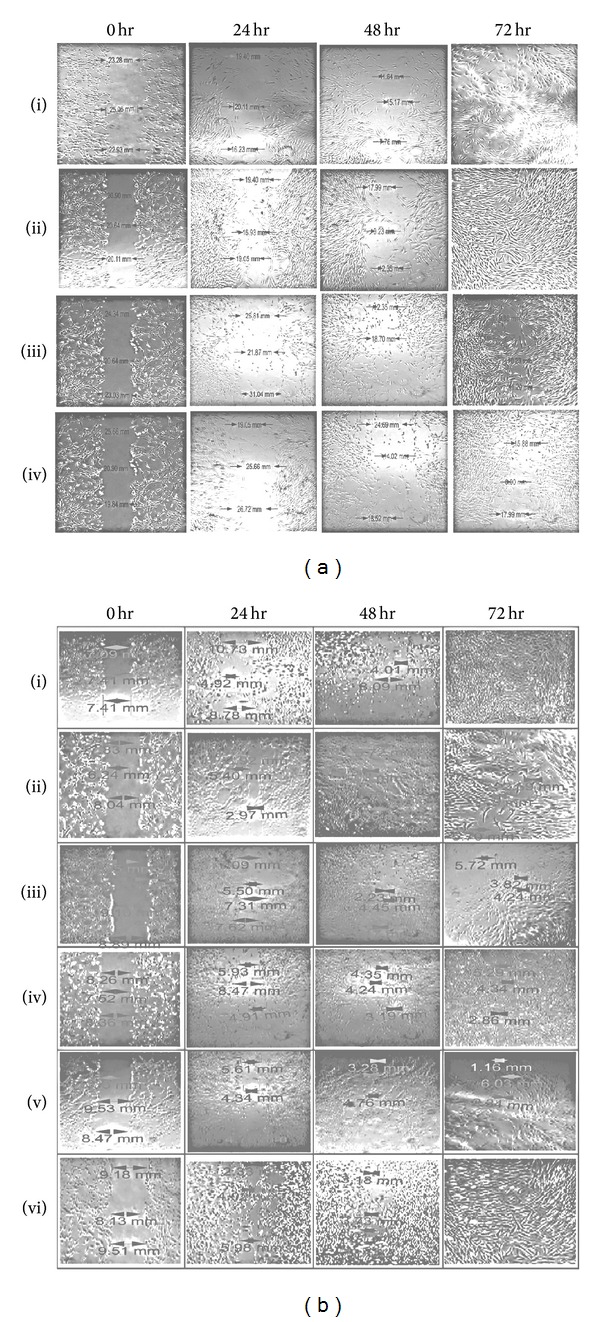
Digital image showing the effect of different fractions of *M. oleifera *on human dermal fibroblast migration in a wound scratch test assay: (a) (i): control without treatment; (ii): methanol; (iii): ethanol; (iv): aqueous extracts; (b) (i): control; (ii): n-hexane; (iii): dichloromethane; (iv): ethyl acetate; (v): n-butanol; (vi): aqueous fractions. A confluent monolayer of human dermal fibroblast (HDF) was scratched using a sterilised 200 *μ*L pipette tip. Different fractions were applied as treatments to the wounded (open gap) and fibroblast media served as control. Migration of fibroblast cells were captured and measured using light microscope attached to a digital camera. Magnification (4x).

**Figure 5:**
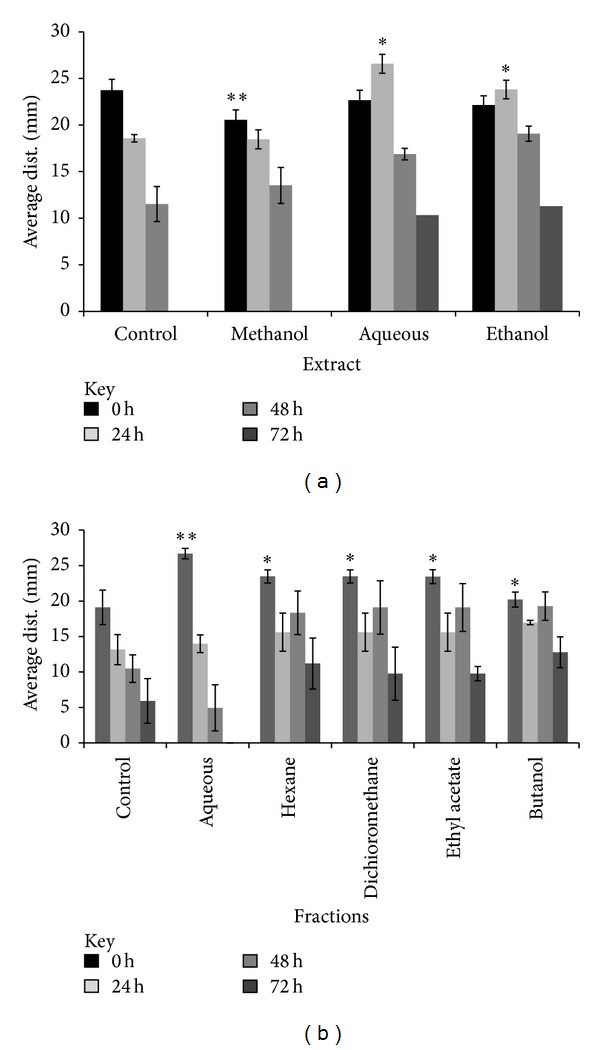
Effect of different crude extracts and fractions of *M. oleifera *on human dermal fibroblast in a wound scratch test. The enhanced migratory HDF cells that completely closed the gap created after 72 hrs were seen in methanolic crude extract and aqueous fraction compared to other solvent extracts. Values are expressed as mean ± SD of triplicate determinations. **P < 0.05 (methanolic extract versus control and other extracts in (a)). **P < 0.05 (aqueous fraction versus control and other fractions in (b)).

The migration of fibroblast cells to cover the scratch created to mimic wound was captured using light microscope attached to a camera, and the distance was measured and analysed quantitatively at time interval of 0, 24, 48, and 72 hrs, after the scratch. The migration of fibroblast was seen to be more significant (*P* < 0.05) in the methanolic crude extract and bioactive aqueous fraction treated cells compared to controls other solvent extracts and fractions (Figures 4(a) and 4(b) and Figures 5(a) and 5(b)). 

### 3.4. Cell Proliferation and Viability Analysis

**Figure 6:**
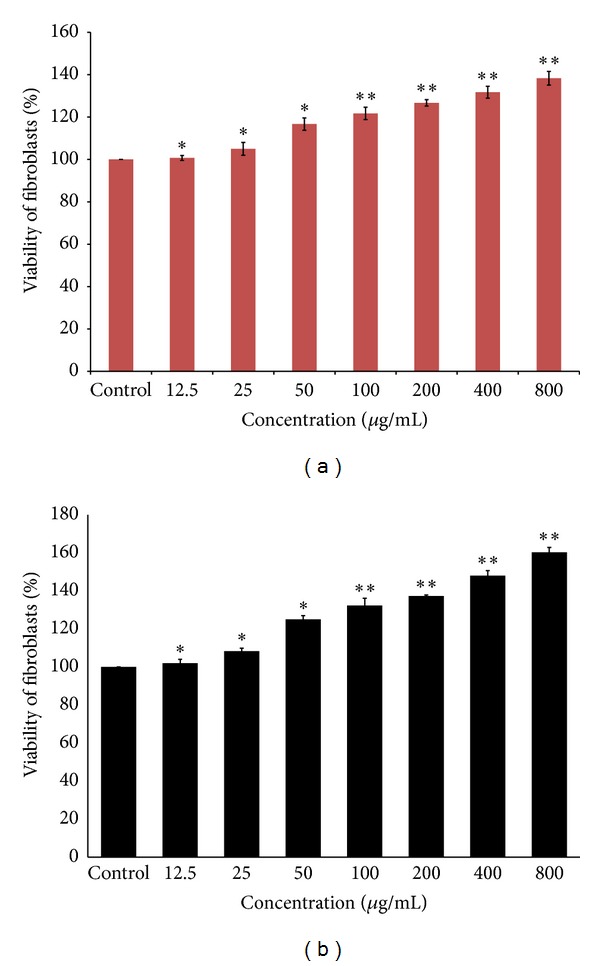
Effects of aqueous fraction treated human dermal fibroblasts (HDF) on cell proliferation and viability of various concentrations at 48 (a) and 72 hrs (b), respectively. The value from baseline control group was set at 100%. The proliferation activity was estimated by MTT assay and calculated by comparing the values from the aqueous fraction treated group with that of control group. Data were expressed as mean ± SD of triplicate values. **P < 0.05 (aqueous fraction treated groups versus control group).

From our results, aqueous fraction of *M. oleifera* significantly increased proliferation and viability of HDF (*P* < 0.05) at concentrations of 100, 200, 400, and 800 *μ*g/mL after 48 and 72 hrs, treatment. These findings indicated that our aqueous fraction was nontoxic to human dermal fibroblast cells even at 72 hrs since it did not affect the cellular activity of fibroblast cells even at high concentration of 800 *μ*g/mL (Figures 6(a) and 6(b)).

### 3.5. Analysis of Bioactive Compounds in Methanolic Crude Extracts and Aqueous Fraction

**Figure 7:**
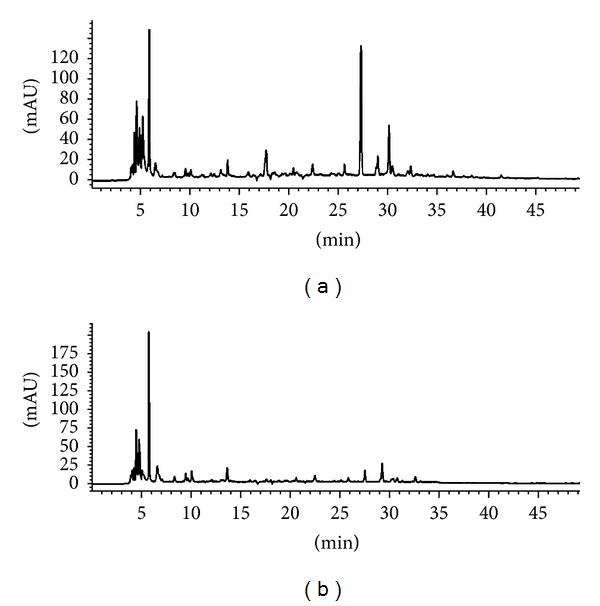
HPLC-DAD chromatogram of (a) methanolic crude extract and (b) aqueous fraction of *M. oleifera*. The peaks represent general profiling of compounds and the pattern of distribution showed a combination of polar, intermediate polar, and nonpolar compounds as represented in methanolic crude extract, while only polar region was observed in aqueous fraction.

The HPLC revealed pattern of distribution of compound present in both methanolic crude extract and its aqueous fraction. The peaks showed the presence of polar and nonpolar compounds in the crude methanolic extract (Figure 7(a)), while only a peak present in the polar region was observed in the aqueous fraction phase (Figure 7(b)). The LC-MS/MS results showed spectral data of major bioactive compounds identified in methanolic crude extract and bioactive aqueous fraction of *M. oleifera* leaves detected with MS in negative and positive modes. The spectral data from the peaks were identical to those of quercetin-3-*O*-glucoside and kaempferol-3-*O*-glucoside in the methanolic crude extract and Vicenin-2 in the aqueous fraction and the identification was based on the LC-MS/MS data and comparison with the literature. The overview is shown in the chromatographic data summarized in Table 1.

### 3.6. Quantification and Confirmation of Bioactive Vicenin-2 from Aqueous Fraction

**Figure 8:**
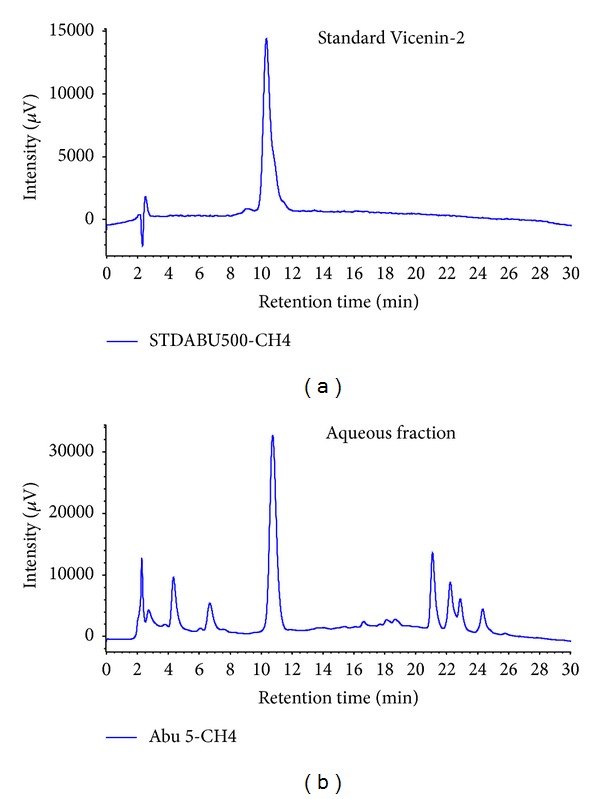
HPLC-DAD chromatogram for reference standard Vicenin-2 (a) and aqueous fraction sample of *M. oleifera* (b). Both peaks have similar retention time of about 11 mins indicating that our aqueous fraction sample contains Vicenin-2 as the major active compound.

The confirmation of Vicenin-2 was carried out using UV/Vis spectroscopic measurement by comparing the HPLC chromatogram of the aqueous fraction sample with that of the standard Vicenin-2. Specificity was established by lack of interfering peaks at the retention time for 11 mins for both sample and the standard (Figures 8(a) and 8(b)). The calibration curves for standard and sample were found to be linear over regression coefficient (*R* ^2^) of 0.988. The accuracy of this analytical method was evaluated by analyzing the sample and standard at different concentrations.

## 4. Discussion

Wound healing occurs as a cellular response to injury and involves activation of fibroblasts, endothelial cells, and macrophages [24]. Fibroblast proliferation is involved in the restoration of structure and function in the wound site [25]. Therefore, therapeutic bioactive agents that are able to stimulate fibroblast growth and proliferation may be able to improve or promote wound healing as in the case of our present study, we demonstrated the effect of crude extract and fractions of *M. oleifera* in enhancing the proliferation and viability of human fibroblast cells in *in vitro*.

According to the observed *in vitro* screening assays, methanolic crude extract significantly caused higher stimulation of fibroblasts compared to ethanolic and aqueous crude extracts. The yield of extract was higher in the methanolic crude extract compared to other solvent extracts, while aqueous fraction yielded higher amount of extract compared to other fractions.

Following differential fractionation of the active methanolic extract, the bioactive aqueous fraction significantly enhanced proliferation and viability of fibroblast cells when compared to untreated control and other solvent fractions, respectively. Therefore, aqueous fraction may be considered as a potential active fractions which contains bioactive agent that enhances *in vitro* wound healing because of its ability to stimulate fibroblast proliferation as reported in earlier studies that fibroblast cell migration and proliferation are essential events for tissue healing and are directly related to its success [26–28].

The morphological changes of fibroblast cells following treatment with crude extracts and fractions as revealed by visual examination with phase contrast microscope clearly demonstrated that methanolic extract treated fibroblast showed higher proliferation rate when compared to other extracts. However, among the different fractions tested, aqueous fraction treated fibroblast showed higher proliferation rate compared to untreated control and other fractions. This may therefore explain in part the potential ability of our aqueous fraction in enhancing wound healing *in vitro*. Our results is in agreement with that of a previous study which reported stimulation of fibroblast by *Buddleja globosa* leaf extract as a wound healing property [29].

The enhancement of fibroblast growth by the methanolic crude extract and aqueous fraction was further justified by estimated cell numbers using the hemocytometer and typan blue dye exclusion assay. The result of this assay also showed that methanolic crude extract treated fibroblast was more viable with increasing cell numbers compared to other extracts, while the aqueous fraction treated fibroblast showed more viable cells compared to untreated control and other solvent fractions.

The methanolic extract being the most bioactive crude extract suggests fully that the compound(s) responsible for the proliferative action may be chemically polar and inorganic because methanol is an inorganic solvent in addition to its polar property. Similarly, aqueous fraction from the methanolic crude extract exhibited high fibroblast proliferative and viability action when compared to other fractions which may be justified by the fact that bioactive compound(s) responsible for the enhancement of proliferation and migration of fibroblast cells may be polar compounds as revealed by the HPLC general profiling results (Figure 7).

The LC-MS/MS analysis of methanolic crude extract and its corresponding aqueous fraction using the coupled UV/MS (negative and positive modes) provides a complete investigation for all the major peaks of the chromatograms. This method has made it possible to identify 2 active compounds: kaempferol and quercetin in the methanolic crude extract of *M. oleifera* and for the first time a flavone C-glucoside compound Vicenin-2, in the aqueous fraction (Table 1). Vicenin-2 was confirmed using HPLC analysis and UV/Vis spectroscopy by comparing the aqueous fraction sample to that of the standard Vicenin-2 compound. In the HPLC analysis, it was observed that both the sample and the standard had a similar retention time of 11 mins and this was further evaluated by spectroscopic measurement. The bands that appeared in the aqueous fraction sample remain between 272 and 338 nm similar to that of the standard Vicenin-2 throughout the reaction period. This result suggested that both the sample and standard have the same characteristic UV-Vis absorption spectrum.

The identified bioactive compounds in the methanolic crude extract and aqueous fraction of *M. oleifera* may serve as a lead in the drug discovery and development of a wound healing agent that may enhance wound healing. These active compounds belong to the group of flavonoid compounds, this group of compounds is known to be beneficial in wound healing treatment, and this has been linked to their antioxidant property [30, 31].

## 5. Conclusion

Our study clearly demonstrated the activity of the crude extracts and fractions of *M. oleifera* in enhancing proliferation and viability of HDF cells in *in vitro*. The bioactive compounds present in aqueous fraction of this plant crude extract have been successfully identified and confirmed by HPLC and LC-MS/MS using reference compound which was procured from commercial source. Aqueous fraction of *M. oleifera* may therefore be a lead in the drug discovery of wound healing active agent. Based on these fruitful findings, further preclinical investigation of the aqueous bioactive fraction and identified bioactive compounds are currently being conducted by our research group.

## Figures and Tables

**Table 1:** LC-MS/MS spectral data of major compounds identified in methanolic crude extract and bioactive aqueous fraction of *M. oleifera* leaves detected with MS in negative and positive modes. The spectral data from the peaks were identical to those of quercetin, kaempferol, and Vicenin-2.

Sample	RT (min)	Mol·wt	M − H	M + H	MZ	UV	Compound identification
Crude extract	**17.82**	464	463 (35%)	465	301	255, 350	Quercetin-3-O-glucoside
	**20.89**	448	447 (35%)	449	285	255, 240	Kaempferol-3-O- glucoside

Aqueous fraction	**12.79**	594	593 (35%)	595	575, 503, 493, 525, 353.	270, 335	Vicenin-2
