# Expression of Concern on “*In Vitro* Wound Healing Potential and Identification of Bioactive Compounds from *Moringa oleifera* Lam”

**DOI:** 10.1155/2020/8687291

**Published:** 2020-11-29

**Authors:** BioMed Research International

BioMed Research International would like to express concern with the article titled “*In Vitro* Wound Healing Potential and Identification of Bioactive Compounds from *Moringa oleifera* Lam,” [1], due to figure duplication.

As noted on PubPeer [2], in Figure 4(a) showing “Digital image showing the effect of different fractions of *M. oleifera* on human dermal fibroblast migration in a wound scratch test assay”, the panels 0 hr (iii) and 0 hr (iv) are highly similar, although they are described as ethanol and aqueous extracts, respectively.

We asked the authors for their explanation and to provide the uncropped and unadjusted images underlying all the figure panels in Figure 4, but we did not receive a response and we are contacting authors' institution to ask them to investigate. This notice may be updated or replaced based on the outcome of the institutional investigation.
